# The Clinical Infection with Pigeon Circovirus (PiCV) Leads to Lymphocyte B Apoptosis But Has No Effect on Lymphocyte T Subpopulation

**DOI:** 10.3390/pathogens9080632

**Published:** 2020-08-03

**Authors:** Tomasz Stenzel, Daria Dziewulska, Bartłomiej Tykałowski, Andrzej Koncicki

**Affiliations:** 1 Department of Poultry Diseases, Faculty Veterinary Medicine, University of Warmia and Mazury in Olsztyn, 10-719 Olsztyn, Poland; daria.pestka@uwm.edu.pl (D.D.); koncicki@uwm.edu.pl (A.K.); 2 Department of Microbiology and Clinical Pathology, Faculty of Veterinary Medicine, University of Warmia and Mazury in Olsztyn, 10-719 Olsztyn, Poland; bartlomiej.tykalowski@uwm.edu.pl

**Keywords:** apoptosis, digital droplet PCR, flow cytometry, lymphocytes, pigeons, pigeon circovirus

## Abstract

The pathology of pigeon circovirus (PiCV) is still unknown, but it is regarded as an immunosuppressant. This study aimed to find a correlation between PiCV natural infection and immunosuppression. The study was conducted with 56 pigeons divided into the following groups: PiCV-positive but showing (group S) or not (group I) non-specific clinical symptoms and asymptomatic pigeons negative for PiCV (group H). The percentage and apoptosis of T CD3^+^ and B IgM^+^ splenocytes; the expression of CD4, CD8, and IFN-γ genes in splenic mononuclear cells; the number of PiCV viral loads in the bursa of Fabricius; and the level of anti-PiCV antibodies were analyzed. The results showed that the percentage of B IgM^+^ cells was almost two-fold lower in group S than in group H, and that ca. 20% of the lymphocytes were apoptotic. No increased apoptosis was detected in TCD3^+^ subpopulation. The PiCV viral loads were approximately one thousand and ten thousand times higher in group S than in groups I and H, respectively. Our results indicate a possible correlation between the number of PiCV viral loads and severity of PiCV infection and confirm that PiCV infection leads to the suppression of humoral immunity by inducing B lymphocyte apoptosis.

## 1. Introduction

One of the most significant infectious agents found in pigeons is the pigeon circovirus (PiCV), which belongs to the genus *Circovirus* and the family *Circoviridae* [1]. The circovirus infection in pigeons was initially documented almost 30 decades ago in Canada, the USA, and Australia [2,3]. Today, PiCV is distributed worldwide with an average prevalence at ca. 70% [4]. Asymptomatic infections with this virus are quite common and were noted in ca. 44% of domestic pigeons, on average, in Poland and in ca. 63% of domestic pigeons in China [5,6,7]. The virus is transmitted mainly horizontally through ingestion or inhalation of virus-contaminated fecal material and feather dust [8]. The high PiCV prevalence results from the specifics of pigeon breeding and rearing system. Bird racing, pigeon exhibitions, and “one loft race” breeding facilities could contribute to the rapid spread of PiCV infections in pigeon populations and to the production of recombinant variants of the virus, as has been noted for other avian circoviruses such as those infecting parrots [9]. The recombination events often detected in pigeon circovirus genome could result from the procedures mentioned above [5,10,11].

The pathology of PiCV infections in pigeons has not been fully investigated to date. PiCV mainly targets the bursa of Fabricius (bF), but its genetic material has also been detected in other organs associated with the immune system, including the thymus and the spleen. The virus or its genetic material have also been detected in various organs of the digestive tract and also in the skin, thyroid gland, and third eyelid [5,12,13]. Pigeon circovirus infections have led to the loss of lymphoid tissue in immune system organs, and for this reason, PiCV is regarded as an immunosuppressive agent in pigeons [14,15]. A higher prevalence of accompanying infections with various pathogens in PiCV-positive pigeons suggests that this virus could induce immunosuppression [15,16,17]. The mechanism of immunosuppression induced by PiCV has not been thoroughly elucidated, but it appears that PiCV infections cause lymphocyte damage [15]. The porcine circovirus type 2 and duck circovirus can activate lymphocyte apoptosis [18,19], and a similar mechanism could be involved in PiCV infection. The laboratory protocol for PiCV culturing has not been developed yet, which is why there is no possibility of performing an experimental challenge with this virus. The above makes performing experiments with PiCV difficult, but investigations with pigeons naturally infected with the circovirus are a possible alternative [20].

This study aimed to answer whether there is any correlation between PiCV natural infection and immunosuppression and, if yes, which main mechanisms of immunity are impaired by the virus.

## 2. Results

### 2.1. Flow Cytometry Analyses

#### 2.1.1. The Percentage of T CD3^+^ and B IgM^+^ Lymphocytes in the Spleen

**Figure 1:**
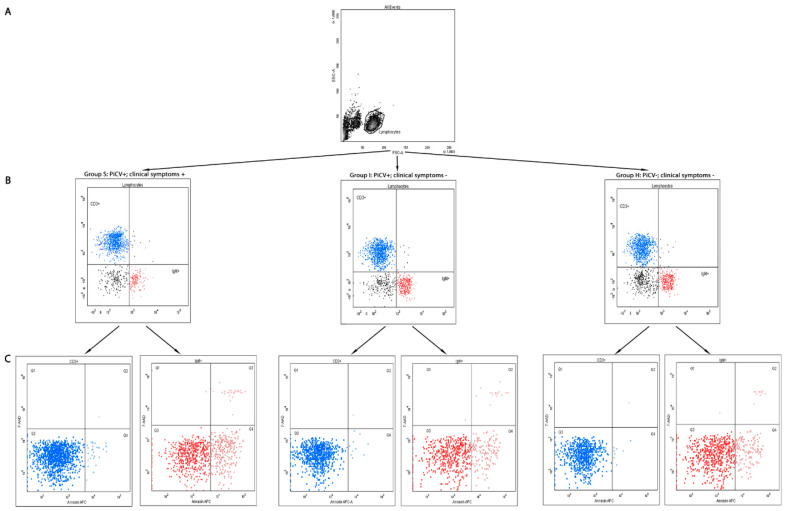
Representative figures of results of the flow cytometry of mononuclear cells isolated from the spleen of pigeons with different PiCV infectious status: (**A**) the lymphocyte gate; (**B**) density plots of the extracellular staining for T CD3^+^ and B IgM^+^; (**C**) density plots of the Annexin V and 7-AAD staining. Three states of the cells were identified: viable cells, located lower left/Q3 (Annexin V-APC/ 7-AAD = −/−); early apoptotic cells, located lower right/Q4 (Annexin V-APC/ 7-AAD = +/−); necrotic and late apoptotic cells, located upper part/Q1 and Q2 (Annexin V-APC/ 7-AAD = −/+ and +/+). The samples were standardized to 1 × 10^6^ of mononuclear cells.

Representative figures of all cytometric analyses of splenic mononuclear cells isolated from the examined pigeons are shown in Figure 1.

**Figure 2:**
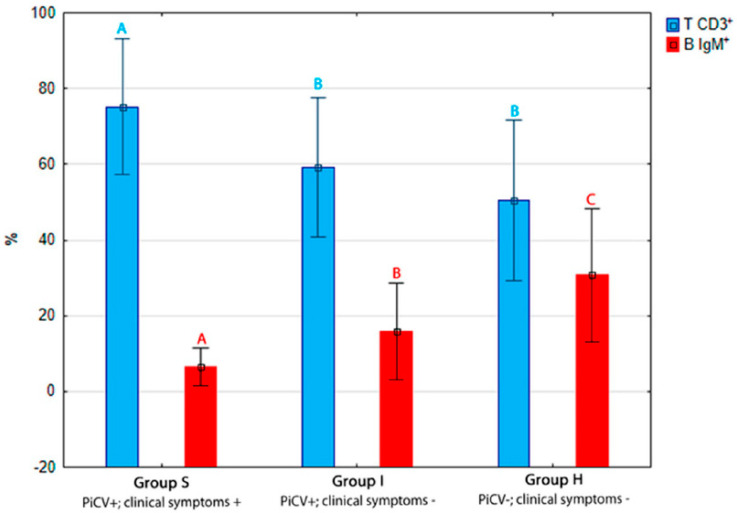
The results of the flow cytometry analyses. The samples were standardized to 1 × 10^6^ of mononuclear cells. The mean percentage and the ratio of T CD3+ to B IgM+ splenic lymphocytes isolated from the examined pigeons. The different letters (^A,B,C^) indicate a statistically significant difference between investigated groups (*p* < 0.01 and *p* < 0.05, respectively) in the Kruskal–Wallis non-parametric test for independent samples. Error bars represent the standard error of the mean.

The average percentage of TCD3^+^ lymphocytes was the highest in the spleen samples isolated from group S pigeons (75.22 +/− 8.92%). The values of this parameter differed statistically (*p* = 0.00) between birds from group I (59.05 +/− 9.20%) and group H (50.45 +/− 10.56%). In turn, the average percentage of splenic B IgM^+^ cells was the highest in the control group (H) pigeons, reaching 30.68 +/− 8.82%, and differed statistically (*p* = 0.00) between groups I (15.81 +/− 6.38%) and S (6.55 +/− 2.44%). The percentage of this lymphocyte subpopulation also differed between pigeons from groups S and I (*p* = 0.00) (Figure 2).

#### 2.1.2. Apoptosis and Necrosis in Splenic T and B Lymphocytes

The percentage of apoptotic T CD3^+^ cells was the highest in group H pigeons (4.33 +/− 1.25%) and differed (*p* = 0.02) only compared to the birds from group I (2.60 +/− 0.96%). There was no statistically significant difference in apoptotic TCD3+ cell percentage between birds from group S (3.00 +/− 1.56%) and the other two groups.

**Figure 3:**
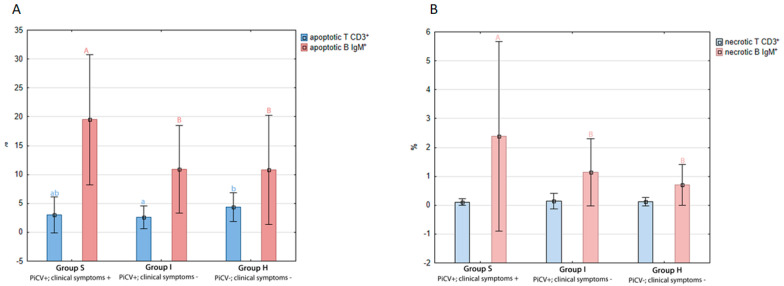
The results of the flow cytometry analyses. The samples were standardized to 1 × 10^6^ of mononuclear cells. (**A**) The mean percentage and the ratio of apoptotic (early apoptosis) T CD3^+^ to B IgM^+^ splenic lymphocytes isolated from the examined pigeons. (**B**) The mean percentage and the ratio of necrotic and late apoptotic T CD3^+^ to B IgM^+^ splenic lymphocytes isolated from the examined pigeons. The different letters (^A,B,C^ or ^a,b^) indicate a statistically significant difference between investigated groups (*p* < 0.01 and *p* < 0.05, respectively) in the Kruskal–Wallis non-parametric test for independent samples. Error bars represent the standard error of the mean.

The percentage of apoptotic B IgM^+^ in group S pigeons reached 19.51 +/− 5.63% and was statistically different (*p* = 0.00) from the respective values noted in birds from group I (10.90 +/− 3.79%) and H (10.78 +/− 4.71%) (Figure 3A).

There were no statistically significant differences in the percentage of necrotic T CD3^+^ cells between all the investigated groups of pigeons. The average percentage of this splenocyte subpopulation was at 0.13 +/− 0.11%.

The highest percentage of necrotic B IgM^+^ cells was detected in group S pigeons and reached 2.38 +/− 1.64%. It differed statistically (*p* = 0.00) from the values reported in groups I (1.14 +/− 0.58%) and H (0.70 +/− 0.35%), while there were no statistically significant differences between these two latter groups (Figure 3B).

### 2.2. The Expression of CD4, CD8, and IFN-γ Genes

**Figure 4:**
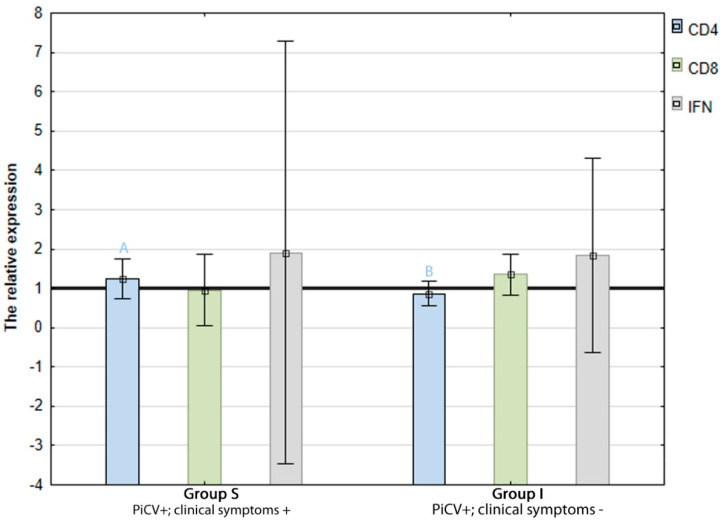
Mean relative expression of the genes encoding CD4 and CD8 lymphocyte receptors and IFN-γ in splenic mononuclear cells of the examined pigeons. The mean relative expression values above 1 (horizontal black line) in groups S and I indicate a higher gene expression compared to the control group (H). The different letters (^A,B^) indicate a statistically significant difference between investigated groups (*p* < 0.01) in the Mann–Whitney U test. Error bars represent the standard error of the mean.

The mean relative expression of all analyzed genes in both infected groups was similar to that found in the control group (H). The highest expression was detected in the case of the IFN-γ gene in splenocytes isolated from group S pigeons (1.90 +/− 2.68). However, the difference was not statistically significant due to a high standard deviation. The expression of the CD8 gene was insignificantly higher in the splenocytes isolated from group I pigeons (1.34 +/− 0.45) than group S birds (0.94 +/− 0.26). In turn, the expression of CD4 gene was statistically higher (*p* = 0.00) in the lymphocytes isolated from the spleens of group S pigeons (1.23 +/− 0.24) than in those isolated from the group I birds (0.86 +/− 0.24) (Figure 4).

### 2.3. Determination of Anti-PiCV IgY

**Figure 5:**
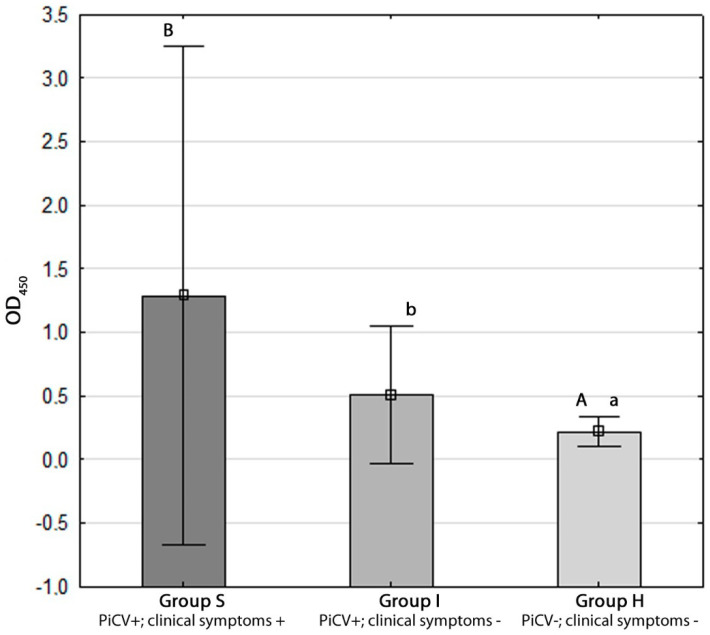
Detection of anti-pigeon circovirus (PiCV) IgY in sera of the examined pigeons using in-house ELISA. The different letters (^A,B^ or ^a,b^) indicate a statistically significant difference between investigated groups in the Kruskal–Wallis non-parametric test for independent samples. Error bars represent the standard error of the mean.

The OD_450_ value reached 1.28 +/− 0.97, 0.51 +/− 0.27 and 0.22 +/− 0.05 in groups S, I, and H, respectively. The statistical difference was detected only between group H and the other two groups at confidence levels of 99% and 95% (*p* = 0.00 and *p* = 0.02 for groups S and I, respectively) (Figure 5).

### 2.4. PiCV Viral Loads in the Bursa of Fabricius Samples

**Figure 6:**
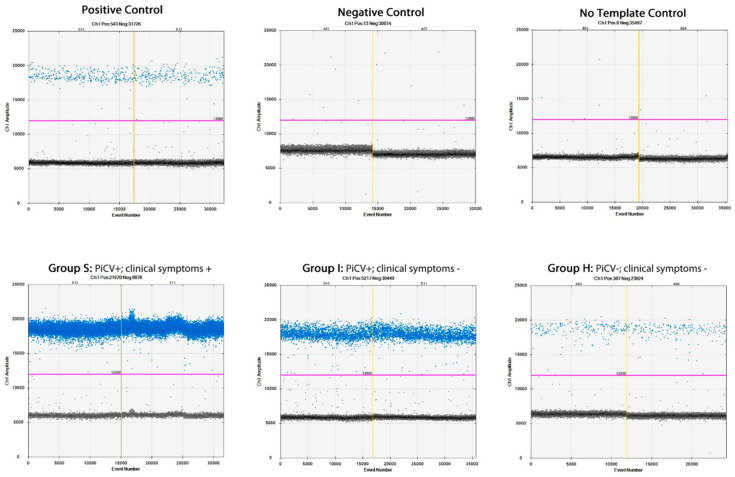
Representative figures of dimensional plots of the ddPCR assay showing PiCV viral loads in the selected samples. The value of y axis (Ch1 amplitude) reflects to amplitude of EvaGreen fluorescence in a detecting channel of a ddPCR device. The sample repetitions are divided by vertical yellow lines. The unbroken pink line is the threshold, above which there are positive droplets (blue) with PCR amplification and underneath which there are negative droplets (gray) without any amplification.

The results of ddPCR for PiCV viral loads are presented in Table 1 and Figure 6. The highest number of PiCV viral loads was detected in the bursa of Fabricius samples of pigeons from group S (36,019,604.61 +/− 66,136,956.68 / 22 µL), and it was statistically different (*p* = 0.00) from PiCV viral loads in the samples of pigeons from group I (38,236.72 +/− 63,281.49 / 22 µL). Surprisingly, the samples of pigeons from group H were also positive, with the lowest PiCV gene copy number (3488.94 +/− 3695.72 / 22 µL). 

## 3. Discussion

Because good-quality racing pigeons are very valuable domestic animals, it is essential to discover mechanisms of the pathology of the most common pathogens occurring in those birds, which is fundamental for the development of proper preventive schedules. One of the most significant infectious agents found in pigeons is the PiCVs. The circoviruses are well known immunosuppressive factors for various animals. The pigeon circovirus is characterized by bursotropism, and the intracytoplasmic inclusion bodies are found mainly in bursal macrophages [13,14,15]. Histologic examination of the bF section positive for PiCV inclusion bodies revealed lymphocyte depletion and necrosis. However, lymphocytic depletion and inclusion bodies were also found in another primary lymphatic organ—thymus. This has underlain a theory that circoviruses could be important factors causing general immunosuppression in pigeons by affecting both T and B lymphocytes [14]. Additionally, one of the previous studies has indicated apoptosis of bursal cells in pigeons positive for PiCV [15]; however, pigeon thymus samples were not investigated with methods allowing the detection of cellular death. One of the methods useful for such analyses is flow cytometry. Its application is feasible because in the early stage of apoptosis, membrane-bound phosphatidylserine is transferred from the cytosol side to the outside of the cell. If fluorochrome-labelled Annexin V is added to the samples, it will create a complex with phosphatidylserine present outside the cells. In this way, the use of Annexin V staining allows distinguishing between viable, necrotic, and apoptotic cells. However, the flow cytometry has some limitations in staining for an apoptosis – the isolated mononuclear cells used for extracellular staining have to be alive. For this reason, every method used for cell isolation should not lead to cellular death. Unfortunately, bF as well as thymus contain high amounts of fibrous connective tissue, and therefore, collagenase needs to be used during mononuclear cell isolation [21]. The treatment with collagenase leads to cellular death, which is why neither bF nor thymus was used for flow cytometry in our study. One of our previous studies has revealed that the secondary immune organ—spleen—could be useful for flow cytometry, because the isolation of splenic mononuclear cells does not require digestion with collagenase, and simple tissue homogenization with the use of a manual grinder is enough. Moreover, high populations of both T and B cells are present in this organ [21,22], and for this reason, we decided to use spleen samples for the isolation of mononuclear cells. 

The results of flow cytometry seem very interesting to us. We detected the significant differences in percentages of both analyzed lymphocyte subpopulations, depending on the PiCV infection status. There were two opposite trends. The percentage of TCD3^+^ lymphocytes increased depending on the PiCV infection severity (PiCV viral loads) and was the highest in group S. In contrast, the B IgM^+^ cells subpopulation was almost two times lower in the birds from group S than in these from group H. The ratio of T CD3^+^ lymphocytes to B IgM^+^ varied from 1.6:1 in group H to 11:1 in group S. Those differences suggest that PiCV infection could affect the B lymphocytes. The Annexin V staining revealed that, despite a difference in group H, apoptosis was at a similar level in T CD3^+^ cells subpopulation isolated from spleens of the examined pigeons. The lack of any trend in the group H birds suggests that this difference could be random and result from the fact that the examined birds originated from different clinical cases, not from experimental inoculation. Much clearer results were present for B IgM^+^ cells. We noted that approximately 20% (two times more than in the subclinically infected pigeons) of those cells were in the early apoptosis state. The mutual proportions between apoptotic T CD3^+^ and B IgM^+^ lymphocytes ranged from 1:2 in group H to 1:6 in group S. Apoptosis is a form of cellular death, which regulates cellular homeostasis by removing unnecessary or damaged cells. The role of virus-induced apoptosis in the lymphocyte depletion and the progression of viral disease has been reported for the best-known animal circovirus—porcine circovirus 2 (PCV-2) [23]. One of the most common biochemical signs of apoptosis is the irreversible fragmentation of genomic DNA, resulting from the activation of nuclear endonucleases that cleave DNA between nucleosomal units. These DNA fragments can be detected at the cellular level on tissue sections by the TUNEL assay, which was performed by Abadie et al. (2001) [15] in pigeons infected with PiCV. The results of those assays partially correspond to ours, because different patterns of lesions (based on apoptosis intensity) were observed in pigeons regarding the PiCV infection status. The number of bF apoptotic cells in sick and PiCV-infected birds was much higher than in the uninfected individuals. Besides, the atrophy of bursal cells was detected. Similar observations were also made for other avian circoviruses like goose circovirus and psittacine beak and feather disease virus [24,25]. Until now, the mechanism of PiCV-induced apoptosis remains unknown. However, in the case of PCV-2, the ORF C3 protein is responsible for lymphocyte depletion and further immunosuppression [26]. The apoptotic activity of ORF 3 protein was also confirmed for duck circovirus [19]. The ORF C3 protein is present in the pigeon circovirus genome [1]; however, its role is still unclear. 

The highest percentage of T CD3^+^ cells in pigeons clinically infected with PiCV suggests the cellular immune response to viral infection. The CD3 receptor is present on all T cells, and, for this reason, we decided to investigate the expression of genes encoding TCD4 and CD8 cellular receptors. Our results showed that the expression of both genes in groups S and I was a slightly higher than in group H. However, those differences were not so apparent like those detected in our previous study [20]. In our opinion, it could be because in the previous study we compared the cellular immune response in PiCV-positive and PiCV-negative pigeons in the context of an additional stimulating factor, which was the immunization with PiCV recombinant capsid protein. However, there is no other literature data to compare our findings. The IFN-γ gene plays a significant role in both immediate and long-term immune responses to a viral infection; hence, its expression was also evaluated in this study. The changes in IFN-γ gene expression were the highest amongst all genes analyzed in this study; however, the result was blurred by massive variation between the samples, especially those collected from pigeons classified as group S. The above could be due to using birds naturally infected with PiCV, which originated from various clinical cases. Those birds were likely to be at different stages of infection. 

A surprising observation was made for anti-PiCV IgY. All of the examined birds were seropositive, which corresponds with the results of our previous study, which revealed that PiCV seroprevalence was ca. 70% regardless of pigeons’ infectious status [6]. The highest antibody levels as well as variability between samples were observed in group S. In this group of pigeons, the values of standard deviation were significantly higher than in the other groups. This phenomenon is typical of investigations conducted with clinical field cases and, similarly like IFN-γ gene expression, suggests that the examined pigeons could be at different stages of infection, which fully corresponds with findings from one of our previous studies [6]. However, due to a lack of possibility of performing experimental infection with PiCV under controlled conditions, experiments conducted with birds originated from clinical cases are the only possible method. One doubt could arise because the B IgM+ lymphocyte percentage was the lowest, whereas the IgY antibody level was the highest in pigeons from group S. It must be remembered, however, that IgY antibodies appear later than IgM. Moreover, the half-life of antibodies may be longer than the life of the lymphocyte. Therefore, with time since infection, the antibody level increases, and the virus-infected cells gradually die.

Until recently, PiCV was considered as one of the putative factors contributing to the complex disease, called Young Pigeon Disease Syndrome, YPDS [27]. This theory was based on the fact that each pigeon presenting symptoms of YPDS was PiCV positive. Moreover, semi-quantitative analyses showed that the amount of PiCV genetic material in bF samples was much higher in the PiCV-positive and diseased birds than in the healthy pigeons [28]. Those observations were partially confirmed by using the qPCR method developed by Duchatel et al. (2009) [29], who revealed that PiCV viral loads were significantly higher in liver samples collected from birds suffering from YPDS than in the subclinically infected individuals. A similar trend was noted for bF and spleen samples, but the differences were not significant, probably because of the small number of examined pigeons. In our study, we decided to use ddPCR for PiCV viral loads quantification, because of its high sensitivity, which allows detecting even single copies of the PiCV genome [20]. The results obtained indicate a strong correlation between PiCV viral loads in bF and the pigeons’ clinical status, because the number of PiCV genome copies was approximately a thousand times higher in the group S pigeons than in the subclinically infected birds. These results correspond to those obtained by the authors mentioned above. Very interesting is also that pigeons classified as group H (negative for PiCV in cloacal swabs and showing no clinical symptoms of YPDS) proved to be positive in ddPCR. The average PiCV viral load was the lowest in this group and was approximately ten times lower than in bF from pigeons classified as subclinically infected. The above indicates that screening pigeons for PiCV based on swab sample examination is not a perfect method and could lead to a false-negative result. However, the swab samples are much better material for PiCV screening than blood samples, which was described earlier [29]. The experimental infection with PiCV performed by Schimdt et al. (2008) [30] has revealed that it is impossible to induce YPDS by pigeon inoculation with this virus and that the combined effect of various factors, like stress factors and virus-induced immunosuppression, could occur. However, since confirmation of rotavirus infection (RVA) in domestic pigeons in Australia, causing the disease similar to YPDS [31], the role of PiCV in the etiology of the disease syndrome has been depreciated. One of the most recent theories says that the characteristic clinical symptoms of the disease are directly related to RVA infection [32,33]. 

Given the current state of knowledge as well as facts found in this study, it should be noted that, despite no evidence for the role of PiCV in the etiology of YPDS, infection with this virus cannot be ruled out as an immunosuppression-inducing trigger. Our results indicate that the pigeon circovirus induces B cell apoptosis, which may affect the impairment of humoral immunity. The above can also explain the slower-developing post-vaccination immunity demonstrated in one of our earlier studies [20]. 

Given the current epizootic situation of PiCV, the intensity of circoviral infections in pigeons showing clinical symptoms of YPDS and the emergence of RVA, it seems reasonable to conduct further studies to determine the real/ potential role of these two viruses in the etiology of YPDS. 

## 4. Materials and Methods 

### 4.1. Ethical Statement

The experiment was carried out in strict observance of the Local Ethics Committee on Animal Experimentation of the University of Warmia and Mazury in Olsztyn (Authorization No. 41/2019). The researchers made every effort to minimize the suffering of birds.

### 4.2. Pigeons

The experiment was conducted in three groups of pigeons. The first group included pigeons naturally infected with PiCV showing non-specific symptoms of the disease (Sick group—S; *n* = 14); the second one included pigeons that were positive for PiCV but clinically healthy (Infected group—I; *n* = 35); and the third one included PiCV-negative pigeons showing no clinical symptoms (control group—H; *n* = 7). All birds were purchased from private breeding facilities. The classification into each group was based on the clinical status of birds and the presence of PiCV genetic material in cloacal swabs. The pigeons showing no less than five of the following clinical symptoms were classified as group S: apathy, decreased body weight, decreased food intake, watery droppings or greenish diarrhea, crop filled with liquid or regurgitation from the crop, increased water intake, extensive diphtheria lesions in beak cavity, respiratory disorders and ruffled feathers. The birds classified as group I showed none of the above clinical symptoms but were positive in PCR screening for PiCV. The pigeons were free of most common pigeon viruses such as avian orthoavulavirus 1, pigeon adenovirus, pigeon herpesvirus, and pigeon rotavirus, which was confirmed by PCR. All investigated birds were young (6–9 weeks old) racing/carrier pigeons. From each bird, blood samples were collected to separate the serum for the detection of anti-PiCV antibodies. Afterward, the birds were euthanized in the CO_2_ chamber, and organs of their immune system (spleen and bursa of Fabricius) were collected for further analyses. 

### 4.3. Isolation of Mononuclear Cells from the Spleen

Mononuclear cells from the whole spleens collected from each bird were isolated with the method described previously [21]. Afterward, the absolute lymphocyte count (ALC) was determined in each sample with a Vi-cell XR automatic cell viability analyzer (Beckman Coulter, Brea, CA, USA). Next, each sample was divided into two parts: the first was used for flow cytometry examination, whereas the second one for RNA extraction for expression of selected genes.

### 4.4. Flow Cytometry Analyses

#### 4.4.1. Extracellular Staining for TCD3^+^ and B IgM^+^ Lymphocytes

The mononuclear cells isolated from spleen samples were standardized to 1 × 10^6^. Next, they were stained for T cells with anti-CD3 (PE) specific monoclonal antibodies obtained in our previous study [21] and for B cells with anti-IgM (FITC) polyclonal antibodies (Biorad, Hercules, CA, USA). After staining, the samples were incubated in darkness on ice for 30 min. Next, the cells were rinsed twice in PBS, centrifuged, and the resulting pellets were resuspended and used for staining for apoptosis evaluation.

#### 4.4.2. Staining for Apoptosis and Necrosis Evaluation

The staining was performed acc. to the protocol described by Maślanka et al. [34]. Cells stained for CD3 and IgM extracellular markers were washed on ice in the Annexin V binding buffer (BD Biosciences, Franklin Lakes, NJ, USA). The supernatants were removed by centrifugation, and the cells were suspended in the same buffer. Next, the APC-conjugated Annexin V (BD Biosciences, Frnaklin Lakes, NJ, USA) and 7-AAD (BD Biosciences, Frnaklin Lakes, NJ, USA) were added to the cells. After incubation, the cells were diluted in the Annexin V binding buffer and analyzed with flow cytometry within 1 h using a FACSCanto II flow cytometer (BD Biosciences, Franklin Lakes, NJ, USA). Data were acquired in FACSDiva Software 6.1.3. (BD Biosciences, Franklin Lakes, NJ, USA). Cells were analyzed and immunophenotyped in FloJo 10.6.2 (BD Biosciences, Franklin Lakes, NJ, USA). Data were expressed as the mean percentage of a particular subpopulation of lymphocytes +/− standard deviation.

### 4.5. RNA Isolation and qPCR for Selected Genes Expression

The number of mononuclear cells isolated from spleen samples was standardized to 5 × 10^6^ and used for RNA isolation. Genomic RNA was isolated using a commercial reagent kit (RNeasy Mini Kit; Qiagen, Hilden, Germany). Reverse transcription was conducted with the commercial kit (High-Capacity cDNA Reverse Transcription Kit; Life Technologies, Carlsbad, CA, USA) according to the manufacturer’s guidelines. The relative expression of the genes encoding CD4 and CD8 T cell receptors and IFN-γ was determined as described previously [22], using a commercial reagent kit (Power SYBR^®^ Green PCR Master Mix kit; Life Technologies, Carslbad, CA, USA) and a LightCycler 96 (Roche, Basel, Switzerland). The relative expression was calculated using the 2^−ΔΔCq^ method [35] normalized to efficiency corrections, average RT and qPCR repeats, control group (H), and reference gene coding glyceraldehyde 3-phosphate dehydrogenase (GAPDH) in GenEx v. 6.1.0.757 data analysis software (MultiD Analyses, Göteborg, Sweden).

### 4.6. In-House ELISA for Determination of Anti-PiCV IgY

The assay was performed according to the protocol described in the previous study [6]. The concentration of detecting antigen was 20 µg/mL, and pigeon sera were diluted at 1:400. The dilution rate of primary antibodies (rabbit anti-pigeon IgG; Antibodies-online, Atlanta, GA, USA) was 1:30,000, whereas the dilution of secondary antibodies (goat anti-rabbit IgG with horseradish peroxidase (HRP); BD biosciences, Franklin Lakes, NJ, USA) was 1:1000. Each rinsing step was performed with an ELx 405 automatic washer (Biotek, Winooski, VT, USA). Optical density was measured with an ELx 800 spectrophotometer (Biotek, Winooski, VT, USA) at the wavelength of 450 nm. Data were expressed as mean OD_450_ +/− standard deviation.

### 4.7. Digital Droplet PCR (ddPCR) for PiCV Viral Loads in the Bursa of Fabricius Samples

The genomic DNA was extracted with the use of DNeasy Blood & Tissue Kit (Qiagen, Hilden, Germany) in accordance with the manufacturer’s instructions. The volume of the tissue homogenate used for DNA extraction was 200 µL. The homogenate was obtained by homogenization (TissueLyser II; Qiagen, Hilden, Germany) of 400 µg of the bursa of Fabricius in 500 µL of the Phosphate Buffered Saline (PBS, Sigma-Aldrich, Schnelldorf Germany). The ddPCR was carried out in a C1000 Touch thermal cycler (Bio-Rad, USA) with the use of ddPCRTM EvaGreen Supermix (Bio-Rad, Hercules, CA, USA). The primer sequences, the composition of the reaction mixture, and thermal cycler conditions were described in our previous study [20]. After amplification, the plate containing the samples was placed in a QX 200 droplet reader (Bio-Rad, Hercules, CA, USA) for analysis. The results were calculated with the following formula: the mean number of positive droplets in the sample minus the mean number of the positive droplets in the negative control (background). The results were expressed as mean PiCV genome copy number +/− standard deviation per 22 µL of the sample.

### 4.8. Statistical Analysis

The statistical analysis of the values of parameters determined in the investigated groups was conducted with the Kruskal–Wallis non-parametric test for independent samples (flow cytometry, ddPCR, in-house ELISA) and Mann–Whitney U test (gene expression) using Statistica.pl v.10.0 software (Statsoft, Kraków, Poland). Differences were considered significant at *p* < 0.05 and *p* < 0.01.

## Figures and Tables

**Table 1:** The PiCV genome copy number in the bursa of Fabricius samples (ddPCR).

PiCV Genome Copy Number/ 22 µL	Group		
	S	I	H
**Min.**	239,794.61	1413.61	533.61
**MN** **SD**	36,019,604.61 A 66,136,956.68	38,236.72 B 63,281.49	3488.94 B 3695.72
**Max.**	215,599,994.60	195,354.61	10,774.61
